# Molecular Mechanisms of Castration-Resistant Prostate Cancer Progression

**DOI:** 10.7759/cureus.83813

**Published:** 2025-05-09

**Authors:** Abdulghani A Naeem, Saud A Abdulsamad

**Affiliations:** 1 Basic Sciences, College of Science and Health Professions, King Saud bin Abdulaziz University for Health Sciences, Jeddah, SAU; 2 Basic Sciences, King Abdullah International Medical Research Center, Jeddah, SAU

**Keywords:** androgen, ar, crpc, degs, kegg, pc3m, pnt2, prostate cancer

## Abstract

Prostate cancer is a global health issue and one of the most common reasons for cancer-related mortality. This research aimed to investigate the molecular mechanisms underlying the progression to castration-resistant prostate cancer (CRPC). Differential gene expression was analyzed by contrasting the PNT2 prostate epithelial cell line and the PC3M CRPC cell line. RNA sequencing was performed on three biological replicates of each cell type, and 1,000 differentially expressed genes were identified with a fold change ≥1 and a p<0.05. A heatmap was generated to visualize the gene expression profiles, and the top 10 significantly altered genes were identified. Functional enrichment analysis, including Gene Ontology and Kyoto Encyclopedia of Genes and Genomes pathway analysis, was conducted to map the biological processes, cellular components, and molecular functions associated with the differentially expressed genes. Furthermore, weighted gene co-expression network analysis was utilized to identify co-expression modules and significant genes, thereby highlighting the top 10 most significant differentially expressed genes in significant pathways. The findings indicate substantial molecular alterations associated with the development of castration-resistant prostate cancer, with major pathways including metabolic deregulation pathways and cell cycle regulation. The identified differentially expressed genes (DEGs) and pathways provide significant insights into disease progression and potential therapeutic targets. These findings contribute to the understanding of prostate cancer at the molecular level and can be used to identify new diagnostic and therapeutic strategies. However, further validation is required to determine the clinical significance of these targets in the treatment of CRPC.

## Introduction

Prostate cancer (PCa) is a worldwide healthcare problem, ranking among the most frequently diagnosed cancers in the male population. It arises in the prostate gland, which is responsible for seminal fluid synthesis. The onset of prostate cancer is influenced by several factors including genetic susceptibility, hormonal impact, and exposure to the environment [1-4]. Importantly, androgens, with particular reference to testosterone, play central roles in the tumorigenesis process as well as in cellular proliferation. In addition, the androgen receptor (AR) is responsible for prostate cancer cell survival, with its activation being a powerful promoter for tumorigenesis [5-7]. Recent studies suggest that pubertal timing may influence prostate cancer risk [8]. A Mendelian randomization study found that delayed puberty may reduce exposure to high androgen levels during prostate development, potentially decreasing prostate cancer risk later in life [9]. This underscores the importance of hormonal regulation in prostate carcinogenesis, highlighting how early-life exposures impact long-term prostate health [10]. As the disease advances, prostate cancer can evolve into castration-resistant prostate cancer (CRPC), a more aggressive form that emerges when tumors no longer respond to androgen deprivation therapies (ADT) [11]. This transition is driven by genetic alterations in key tumor suppressor genes, such as SPOP and BRCA2, which contribute to progression from hormone-sensitive prostate cancer to a castration-resistant phenotype [6,12,13]. Previous research has also uncovered key gene changes linked to the progression of castration-resistant prostate cancer, including mitochondrial adaptations and androgen receptor-independent drivers such as FABP5 [14]. The emergence of CRPC presents a major clinical challenge, as it remains largely incurable and associated with poor patient outcomes. The molecular landscape of prostate cancer is further complicated by the presence of distinct aggressive subtypes, such as neuroendocrine prostate cancer (NEPC) and aggressive variant prostate cancer (AVPC) [14,15]. These subtypes exhibit unique biological behaviors and treatment responses, necessitating the development of targeted therapies and personalized medicine approaches. Identifying biomarkers associated with these aggressive variants is crucial for improving patient stratification and therapy selection [16,17]. This study sought to examine differential gene expression patterns between castration-resistant prostate cancer cells (PC3M) and normal prostate epithelial cells (PNT2), with the aim of elucidating the key molecular mechanisms and signalling pathways underlying the progression to castration-resistant prostate cancer.

Inflammation and the tumor microenvironment in prostate cancer progression

Chronic inflammation is implicated in the onset and progression of prostate cancer. The inflammatory activity in the prostatic tissue in its non-neoplastic state correlates with an increased risk for high-grade prostate cancer [18]. This suggests that proinflammatory mediators may contribute to tumor initiation, necessitating further investigation into the molecular links between inflammation and prostate carcinogenesis [19]. Additionally, the tumor microenvironment (TME), particularly the reactive stroma, plays a pivotal role in prostate cancer progression and metastasis [20]. The interaction between cancer cells and stromal components influences tumor behavior, promoting angiogenesis, immune evasion, and therapy resistance [21]. These findings suggest that the therapeutic approach through the tumor microenvironment may provide new therapeutic targets, especially for CRPC.

Genetic factors and disparities in prostate cancer risk

Genetic susceptibility is a proven risk factor for prostate cancer, and familial clustering of the disease is common. Germline alterations in genes like HOXB13 and BRCA2 have been implicated in hereditary prostate cancer syndromes, highlighting the relevance of genetic testing and counseling in susceptible individuals. Furthermore, racial and ethnic differences in prostate cancer incidence and mortality imply that genetic, environmental, and lifestyle factors all influence disease risk and progression [22]. Understanding these disparities is essential for developing equitable screening strategies and precision medicine interventions.

Study rationale and experimental approach

This study endeavored to elucidate molecular mechanisms involved in prostate cancer by comparing gene expression between CRPC cells (PC3M) and normal prostate epithelial cells (PNT2). PNT2, a prostate epithelial-derived cell line, is commonly used as a control model in research related to prostate cancer. PC3M, originating from a castration-resistant prostate cancer tumor, is a typical castration-resistant phenotype given its ability to grow despite androgen deprivation therapy. This hallmark reflects advanced-stage prostate cancer. Through these two cell lines, the current study provides an important comparison to determine molecular alterations unique to the development of (CRPC) progression. One significant question in CRPC research is whether AR remains the major driver as an oncogene or if other pathways become activated in therapy-resistant tumors. To explore the issue, RNA-sequencing analysis was carried out in three biological replicates from each type of cell, which led to the construction of large gene expression profiles. Altogether, 1,000 genes with differing expressions, referred to as differentially expressed genes (DEGs), were identified based on a cut-off criterion set by a ≥1-fold change (FC) in their expressions with a p<0.05, as analyzed by Student's t-test. The identified DEGs were depicted in a heatmap, which displayed the most significantly up-regulated and down-regulated genes.

Gene ontology, pathway enrichment, and network analysis

Following the announced (DEGs), gene ontology (GO) enrichment analysis grouped these affected genes into certain biological processes, cellular components, and molecular functions, providing insights into gene alterations in cancer progression. Moreover, Kyoto Encyclopedia of Genes and Genomes (KEGG) pathway analysis mapped out signaling pathways enriched in prostate cancer cells, providing an extensive outline of oncogenic signaling networks. To explore functional gene interaction, weighted gene co-expression network analysis (WGCNA) was used, allowing the discovery of modules consisting of co-expressed genes. Hierarchical clustering dendrogram grouped genes with similar expressions, with dynamic tree cutting used to outline co-expression modules, as represented by distinct color in the dendrogram. By uncovering hub genes in relevant modules, this study highlights the importance of key regulatory genes responsible for the development of castration-resistant prostate cancer (CRPC).

Significance and implications

The findings of this study have far-reaching clinical implications. By pinpointing key differentially expressed genes (DEGs), this research identifies potential biomarkers for early prostate cancer detection, which is critical for improving treatment outcomes. Additionally, the DEGs and enriched pathways may serve as novel drug targets, facilitating the development of precision medicine strategies. Targeted therapies addressing these molecular alterations could lead to more effective, personalized treatments with fewer side effects than conventional approaches.

## Materials and methods

Cell lines and culture

The study utilized two prostate cell lines as follows: the PNT2 prostate epithelial cell line, established from a donor without a history of prostate disease, and the highly aggressive, androgen receptor-negative PC3M prostate cancer cell line. Both cell lines were purchased from the American Type Culture Collection (ATCC). These cells were cultured as monolayer cultures at 37°C in a 5% CO_2_ atmosphere, using RPMI-1640 medium supplemented with L-glutamine, penicillin, streptomycin, and 10% fetal bovine serum. All experiments were performed using three independent biological replicates for each cell line. To minimize technical variability, cells were cultured under identical conditions using standardized passage numbers, medium batches, and plating densities.

RNA extraction, RNA library preparation, and NovaSeq sequencing

Total RNA was extracted from the frozen cell pellets using a Qiagen RNeasy Mini kit (Hilden, Germany: QIAGEN N.V.), in accordance with the manufacturer's protocol. The Qiagen FastSelect rRNA HMR kit (Hilden, Germany: QIAGEN N.V.) was employed to prepare the rRNA depletion sequencing library. Additionally, a NEBNext Ultra II RNA Library Production kit (Ipswich, MA: New England Biolabs) for Illumina was used to construct the RNA sequencing library. The libraries were verified using a Tapestation 4200 kit (Santa Clara, CA: Agilent Technologies, Inc.), quantified with a Qubit 2.0 Fluorometer (Waltham, MA: Thermo Fisher Scientific Inc.), and validated by quantitative PCR. Each sample achieved an average sequencing depth of approximately 30 million reads. Quality control was performed before and after alignment using FastQC (Cambridge, UK: Babraham Bioinformatics, Babraham Institute) and MultiQC (Stockholm, Sweden: Philip Ewels), with all samples achieving >90% Q30 scores and no significant contamination or adapter presence. The multiplexed sequencing libraries were then sequenced on the Illumina NovaSeq 6000 System (San Diego, CA: Illumina, Inc.), utilizing a 2×150 Pair-End Configuration, version 1.5 (San Diego, CA: Illumina, Inc.). The raw sequence data generated by the Illumina NovaSeq platform was converted into FASTQ files using the Illumina bcl2fastq (San Diego, CA: Illumina, Inc.) application version 2.20, and subsequently de-multiplexed based on the index sequence identification. The RNA sequencing data analysis involved the following steps: Trimmomatic was employed to trim the sequence reads, remove adapter sequences, and exclude low-quality nucleotides. Trimmomatic (v0.39) was run using the following parameters: sliding window=4:20, minimum read length=36 bp, and removal of Illumina adapters.

Statistical analysis

The data in this study were analysed using GraphPad Prism 9 software (San Diego, CA: GraphPad Software). Two-tailed paired Student's t-tests and one-way ANOVAs were employed to statistically compare group means, where appropriate. For the RNA-sequencing data, differentially expressed genes were identified using the DESeq2 package (Heidelberg, Germany: European Molecular Biology Laboratory {EMBL}), which internally applies the Benjamini-Hochberg procedure to adjust for multiple testing and control the false discovery rate. Genes exhibiting a fold change of 1 or greater and an adjusted p-value less than 0.05 were considered statistically significant. These widely accepted thresholds were adopted in the transcriptomic analyses to strike a balance between sensitivity and specificity in detecting biologically meaningful changes in gene expression [23].

## Results

Differential expression between PNT2 and PC3M

**Figure 1:**
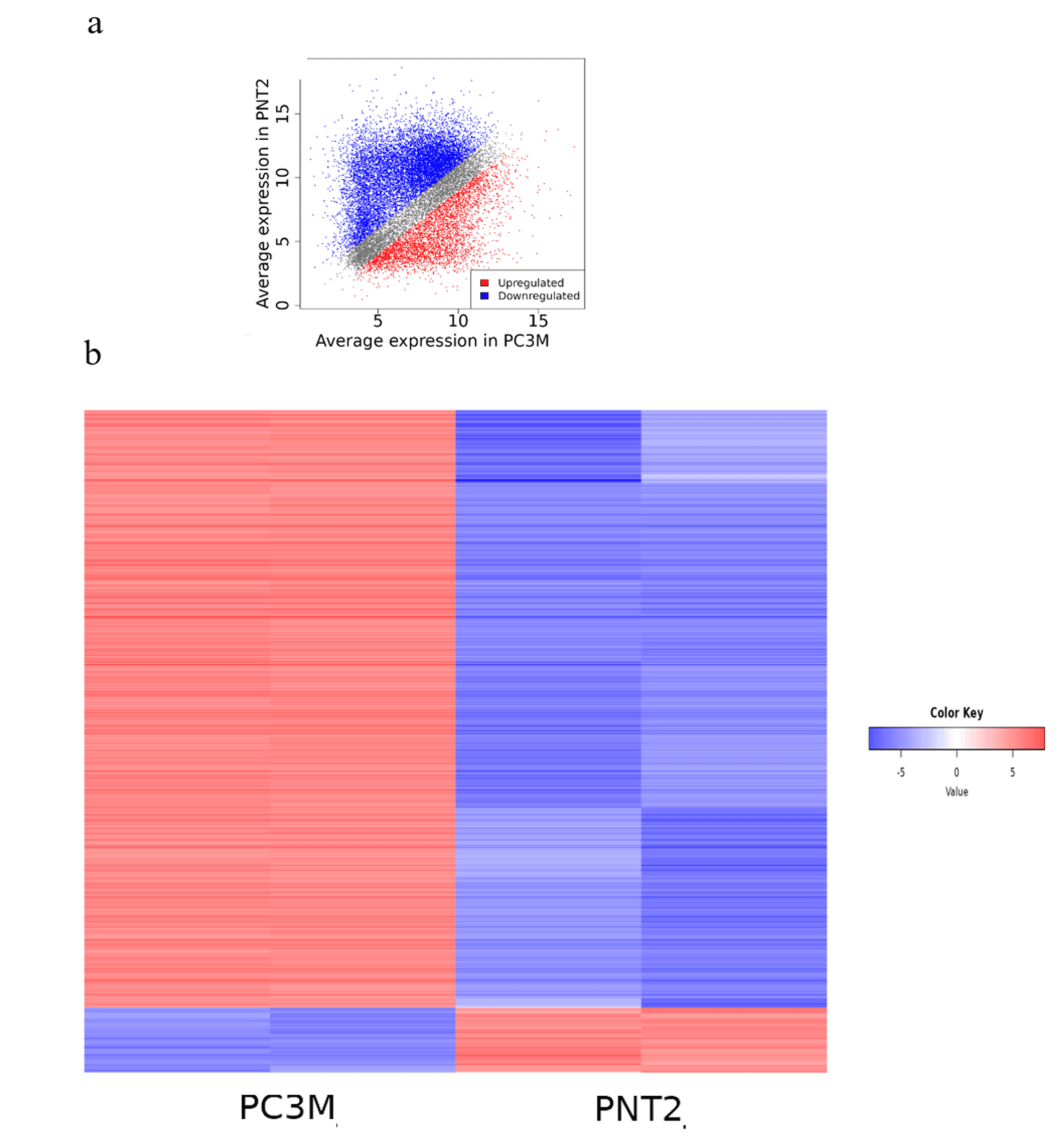
Scatterplot and differentially expressed genes between PNT2 and PC3M cell. (a) Scatterplot depicts the differentially expressed genes (DEGs) profile between the PNT2 prostate epithelial cell line and the PC3M castration-resistant prostate cancer cell line. Genes that are upregulated in the PC3M cells are shown in red, while downregulated genes are represented in blue. This analysis has identified 1,000 differentially expressed genes between the two cell types. (b) Heatmap depicts the expression patterns of the identified DEGs, with upregulated genes in PC3M cells shown in red and downregulated genes in blue compared to the PNT2 cell line.

The scatter plot and differentially expressed genes (DEGs) between PNT2 and PC3M cells are presented in Figures 1a, 1b. Based on an analysis of the scatter plot and RNA-seq heatmaps, the top 1,000 most significant DEGs (500 upregulated and 500 downregulated) were identified (Figures 1a, 1b). These results reveal substantial transcriptional differences between normal prostate epithelial cells (PNT2) and castration-resistant prostate cancer cells (PC3M), suggesting key molecular alterations associated with prostate cancer progression.

Top 10 differentially expressed genes

**Figure 2:**
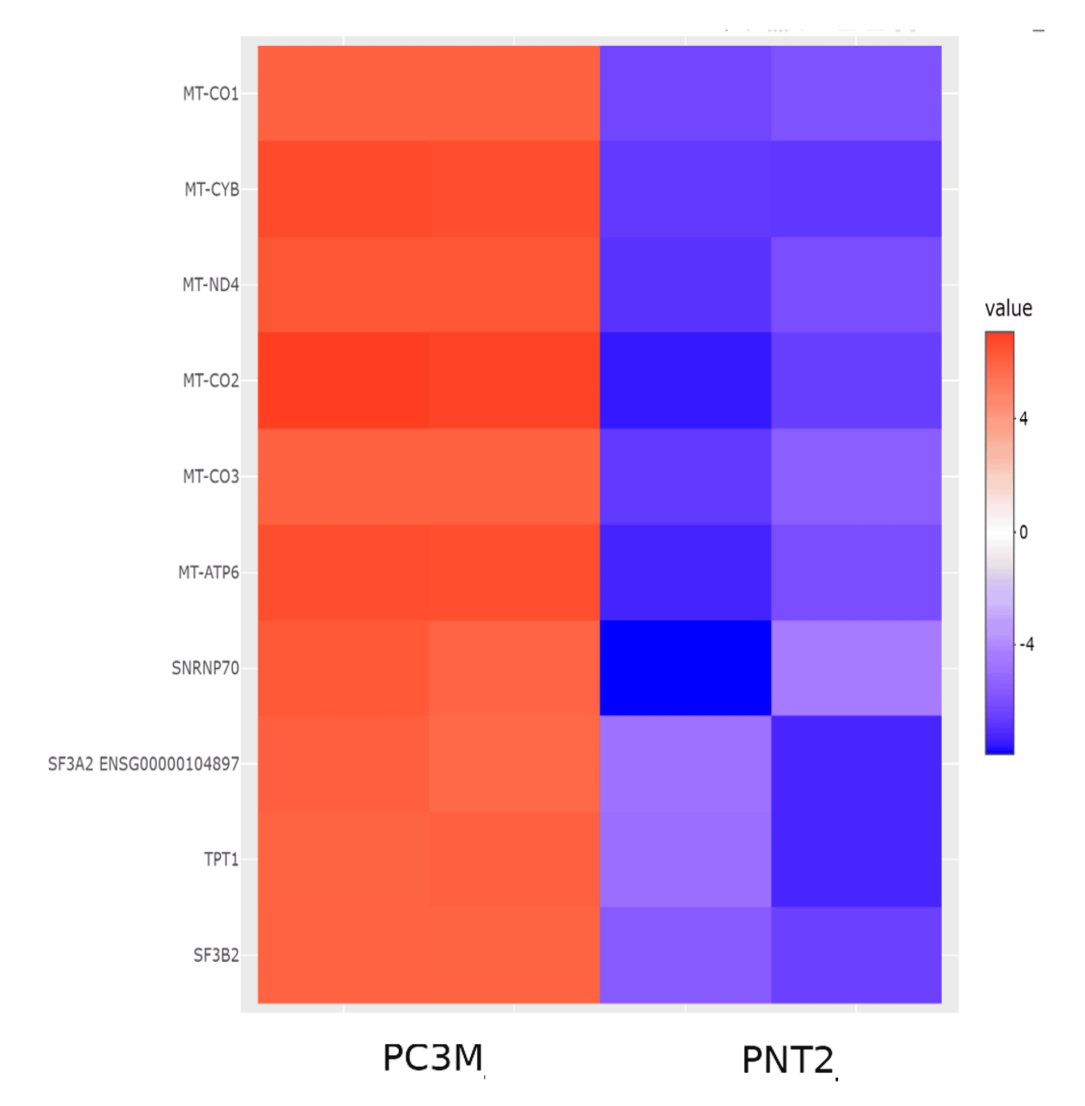
Top 10 DEGs between the PC3 and PNT2 prostate cancer cell lines. Heatmap depicts the expression profiles of the 10 most DEGs between the PC3M castration-resistant prostate cancer cell line and the PNT2 normal prostate epithelial cell line. Upregulated genes in PC3M are represented in red, while downregulated genes are shown in blue in comparison to PNT2.

A comparison of gene expression patterns between PNT2 and PC3M cells identified the 10 most significantly upregulated and downregulated genes, which are presented in Figure 2. The top 10 DEGs in PC3M cells include MT-CO1, MT-CYB, MT-ND4, MT-CO2, MT-CO3, MT-ATP6, SNRNP70, SF3A2, TPT1, and SF3B2, all of which were significantly upregulated in PC3M and downregulated in PNT2 (Figure 2 and Table 1). These findings highlight key mitochondrial and splicing-related genes that may contribute to castration-resistant prostate cancer (CRPC) progression and survival mechanisms.

**Table 1:** List of the 10 most significantly differentially expressed genes (DEGs) between PNT2 and PC3M.

Gene ID	Gene name	Log_2_ fold change	PNT2	PC3M
ENSG00000198804	MT-CO1	402370.8776	77.73854636	402370.8776
ENSG00000198727	MT-CYB	118563.7544	9.893996809	118563.7544
ENSG00000198886	MT-ND4	215952.0172	21.20142173	215952.0172
ENSG00000198712	MT-CO2	164826.8557	5.653712463	164826.8557
ENSG00000198938	MT-CO3	112731.657	15.54770927	112731.657
ENSG00000198899	MT-ATP6	147171.6308	8.480568694	147171.6308
ENSG00000104852	SNRNP70	18850.87334	0	18850.87334
ENSG00000104897	SF3A2	10509.64961	4.240284347	10509.64961
ENSG00000133112	TPT1	29296.58454	14.13428116	29296.58454
ENSG00000087365	SF3B2	21312.50256	5.653712463	21312.50256

GO and pathway analysis

**Figure 3:**
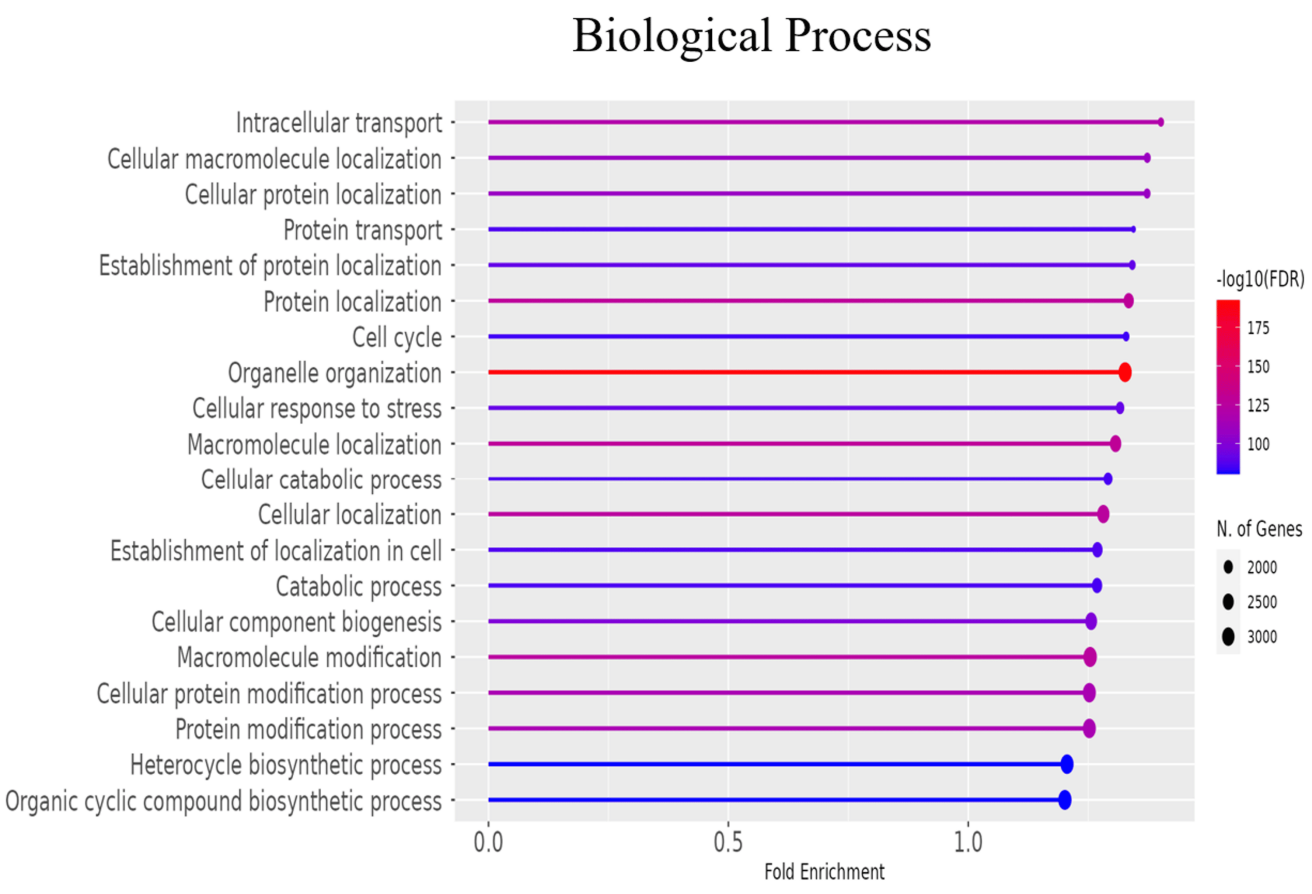
Biological processes analysis. Gene ontology (GO) enrichment analysis revealed the biological processes associated with the DEGs between the PC3M castration-resistant prostate cancer cell line and the PNT2 normal prostate epithelial cell line. The x-axis depicts the fold enrichment, while the y-axis lists the enriched biological processes. The colour gradient represents the adjusted p-value (-log_10_), and the dot size indicates the number of genes involved in each process. FDR: false discovery rate; DEG: differentially expressed gene

**Figure 4:**
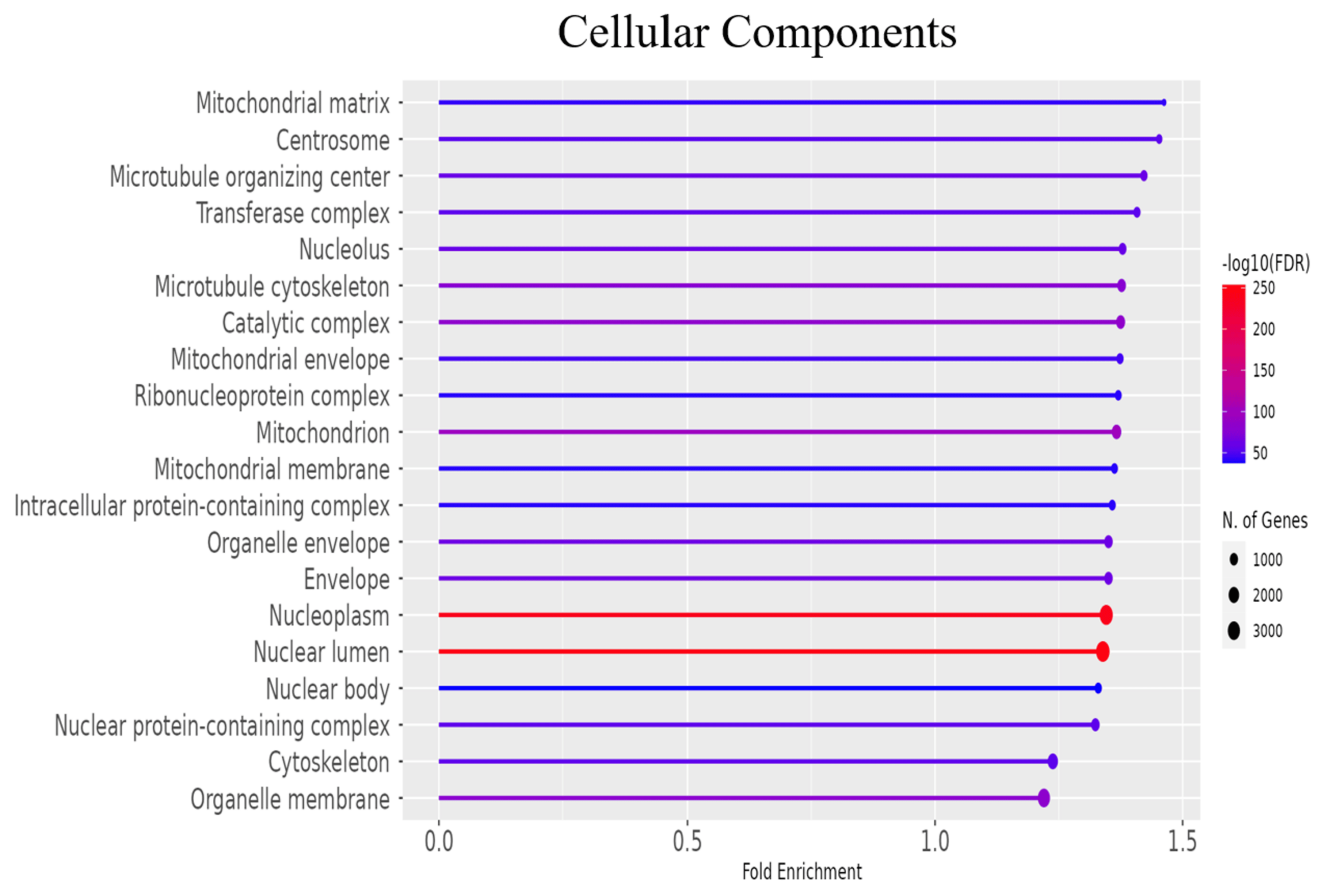
Cellular components analysis. Gene ontology (GO) enrichment analysis revealed the cellular components associated with DEGs between PC3M and PNT2 cells. The x-axis depicts the fold enrichment, while the y-axis lists the enriched biological processes. The colour gradient represents the adjusted p-value (-log_10_), and the dot size indicates the number of genes involved in each process. FDR: false discovery rate; DEG: differentially expressed gene

**Figure 5:**
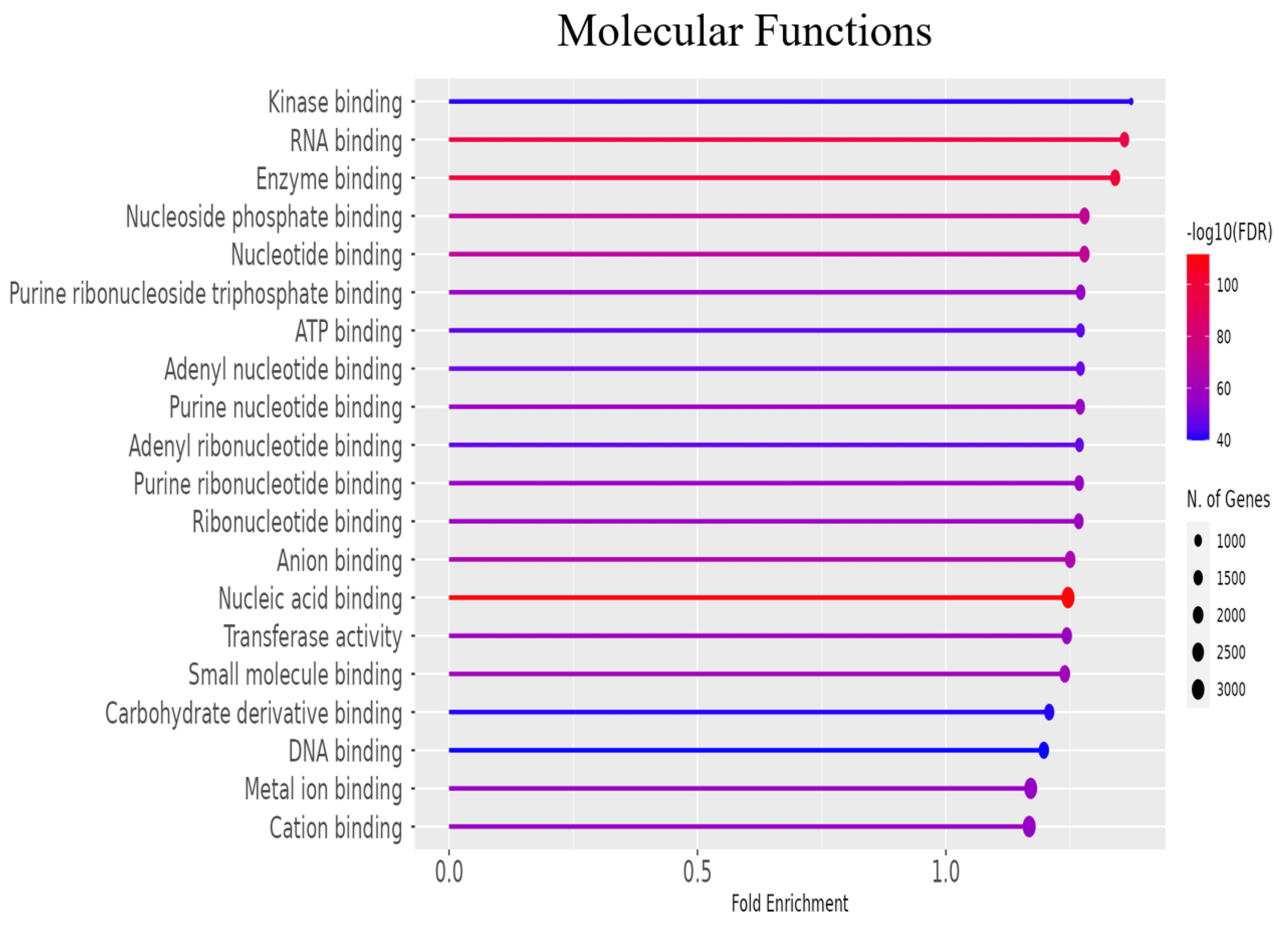
Molecular function analysis. Gene ontology (GO) enrichment analysis revealed molecular functions of DEGs between PC3M and PNT2 cells. The x-axis depicts the fold enrichment, while the y-axis lists the enriched biological processes. The colour gradient represents the adjusted p-value (-log_10_), and the dot size indicates the number of genes involved in each process. FDR: false discovery rate; DEG: differentially expressed gene

**Figure 6:**
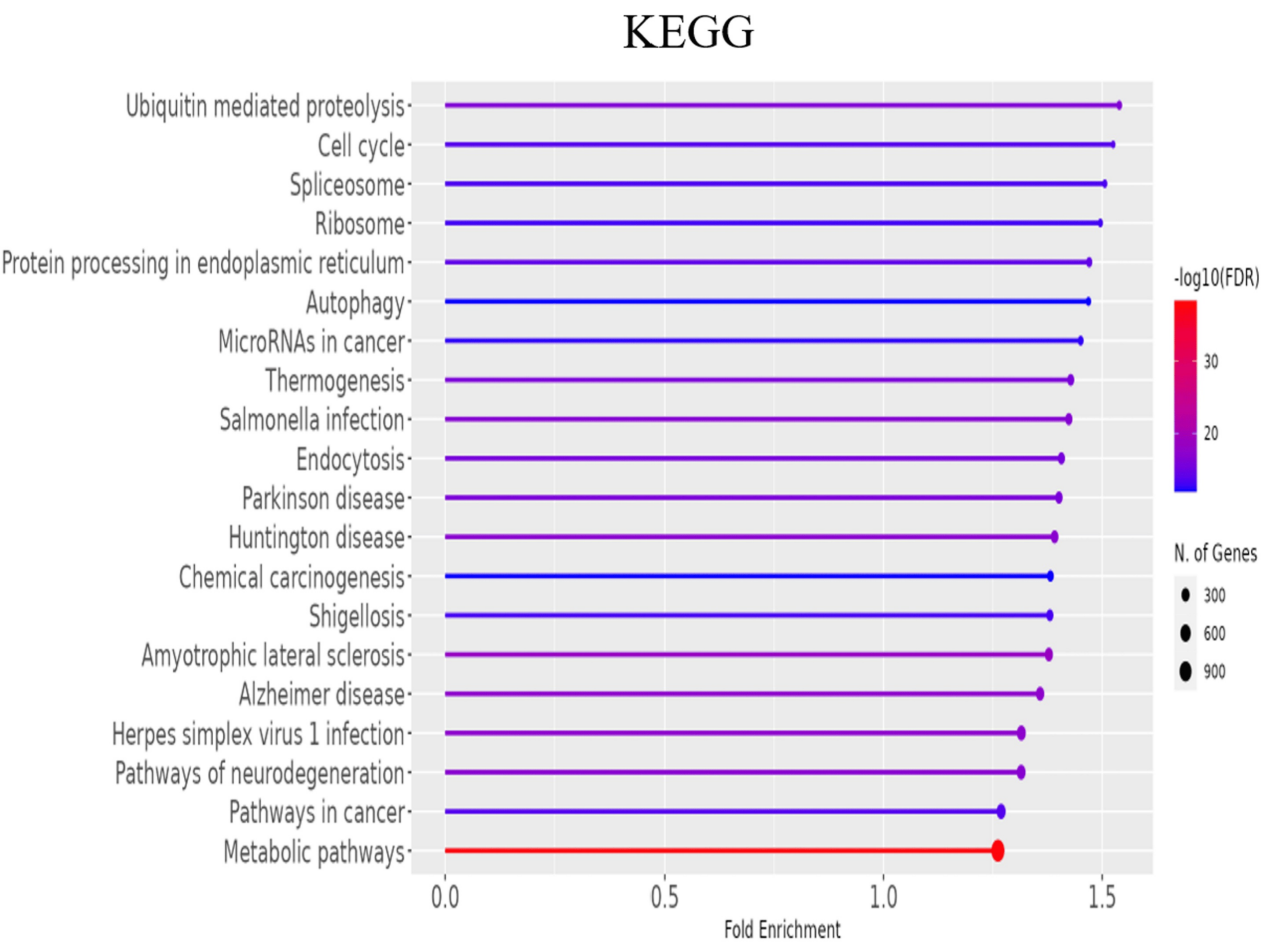
Kyoto Encyclopedia of Genes and Genomes (KEGG) pathway analysis. Kyoto Encyclopedia of Genes and Genomes (KEGG) pathway enrichment analysis of DEGs between PC3M and PNT2 cells. The x-axis represents fold enrichment, while the y-axis lists the significantly enriched pathways. The x-axis depicts the fold enrichment, while the y-axis lists the enriched biological processes. The colour gradient represents the adjusted p-value (-log_10_), and the dot size indicates the number of genes involved in each process. FDR: false discovery rate; DEG: differentially expressed gene

**Figure 7:**
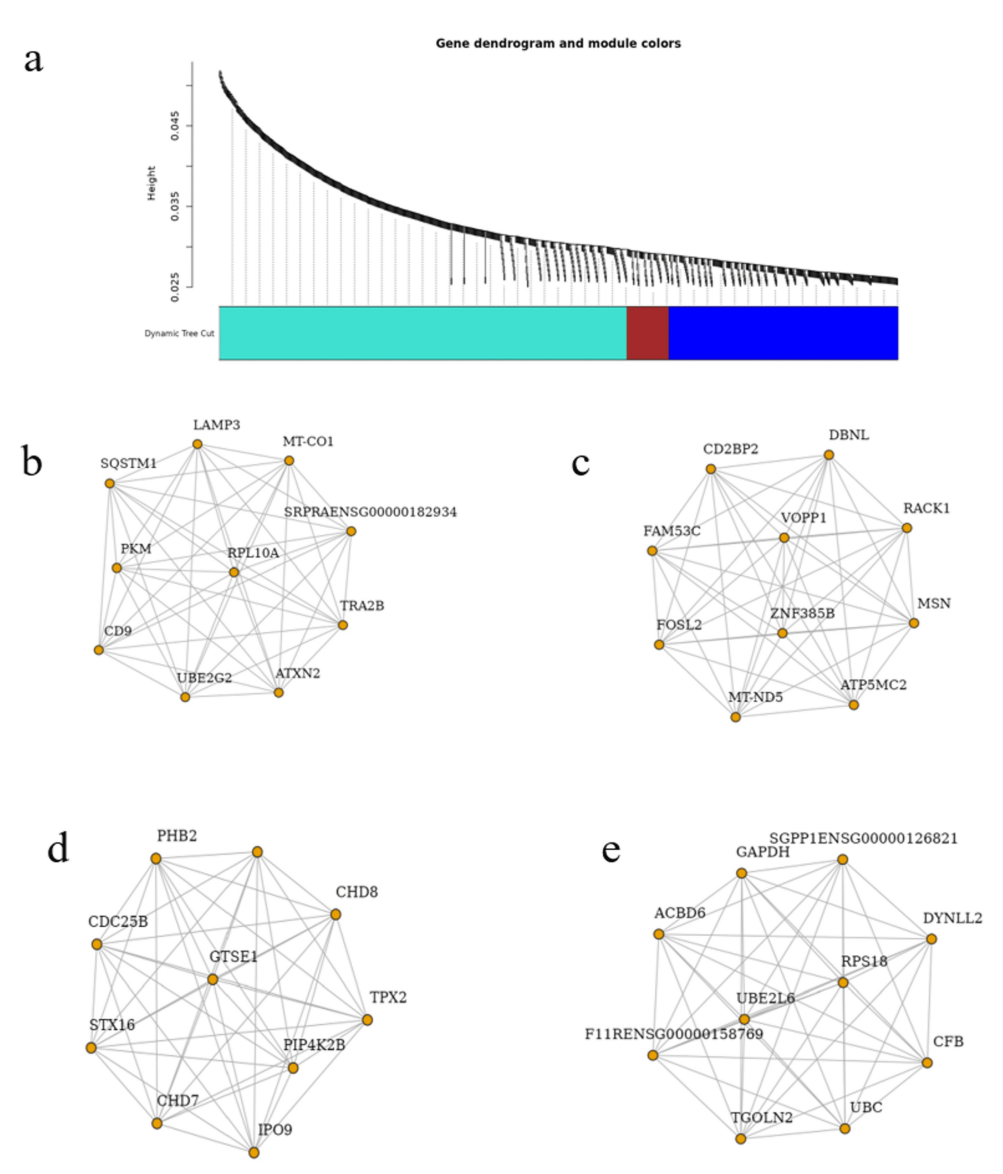
Hub gene and network analysis in prostate cancer. (a) The analysis of differentially expressed genes DEG using hub gene network analysis identified key regulatory genes involved in prostate cancer progression. The bar plot displays the ranked gene significance, with hub genes highlighted in distinct colours based on their connectivity and functional importance. (b-e) Major hub gene interaction networks derived from DEG analysis: (b) A network of genes that regulate cellular communication and signal transduction pathways. (c) A network of genes that regulate metabolic reprogramming and mitochondrial dysfunction pathways. (d) A network of genes that regulate chromatin remodeling and DNA repair pathways. (e) A network of genes that regulates stress response and protein homeostasis. DEG: differentially expressed gene

To further explore the functional roles of the significant DEGs, gene ontology (GO) enrichment analysis was conducted to categorize genes into biological processes, molecular functions, and cellular components (Figures 3-5 and Tables 2-4). Additionally, KEGG pathway analysis delineated the signaling pathways enriched in the DEGs (Figure 6, Table 5). To identify key regulatory genes and potential therapeutic targets, a hub gene network and gene-drug interaction analysis were performed (Figure 7a-7e, Table 6). Analysis in Figure 3 revealed that DEGs were significantly enriched in biological processes such as cell cycle regulation, protein localization and transport, intracellular signaling, macromolecule biosynthesis, metabolic reprogramming, cellular response to stress, and organelle organization. These pathways play essential roles in cancer initiation and progression, promoting uncontrolled proliferation, metabolic shifts, stress adaptation, and oncogenic signaling activation. In Figure 4, GO analysis identified key cellular components associated with the DEGs, including the nucleoplasm, nuclear envelope, nuclear lumen, mitochondria, intracellular protein-containing complexes, and organelle membranes. These components contribute to genomic instability, transcriptional dysregulation, metabolic reprogramming, and structural modifications that facilitate cancer progression and therapeutic resistance. Figure 5 highlights the enrichment of molecular functions, including kinase binding, RNA binding, enzyme binding, nucleotide binding, ATP binding, nucleic acid binding, and transferase activity. These functions support tumor development by enhancing signal transduction, regulating gene expression, driving metabolic changes, and facilitating enzymatic activities critical for sustained proliferation and survival in malignant cells. To explore the signaling pathways activated in the DEGs, KEGG pathway analysis was performed (Figure 6, Table 5). The results demonstrated significant enrichment in pathways related to ubiquitin-mediated proteolysis, cell cycle regulation, spliceosome activity, ribosome function, protein processing in the endoplasmic reticulum, autophagy, microRNAs in cancer, and metabolic pathways. In Figures 7a-7e, further analysis of the differentially expressed genes through hub gene network analysis identified critical regulators involved in cellular communication and signal transduction, metabolic reprogramming and mitochondrial dysfunction, chromatin remodeling and DNA repair, and stress response and protein homeostasis. Figure 7a presents the ranked distribution of the DEGs, while Figures 7b-7e highlight major hub gene interaction networks. Figure 7b reveals genes linked to cellular communication and signal transduction, such as CD9, UBE2G2, and TRA2B. Figure 7c identifies DEGs associated with metabolic reprogramming and mitochondrial dysfunction, including MT-CO1 and ATP5-MC2. Figure 7d highlights key genes involved in chromatin remodeling and DNA repair, such as CHD7, CHD8, and CDC25B. Figure 7e explores DEGs related to stress response and protein homeostasis, including GAPDH and HSP90AA1. The 10 most important hub genes are summarized in Table 6, highlighting their functional roles and biological significance in castration-resistant prostate cancer progression.

**Table 2:** The top 20 GO enrichment analysis of biological process in castration-resistant prostate cancer. FDR: false discovery rate; GO: gene ontology

Enrichment FDR	N genes	Pathway genes	Fold enrichment	Pathway
2.94E-193	3347	4179	1.326855	Organelle organization
1.25E-129	2635	3340	1.306996	Macromolecule localization
4.65E-129	2285	2837	1.334342	Protein localization
2.14E-126	3444	4550	1.253984	Macromolecule modification
3.02E-126	2944	3806	1.281472	Cellular localization
1.82E-120	1571	1857	1.401537	Intracellular transport
7.19E-117	3258	4310	1.252316	Cellular protein modification process
7.19E-117	3258	4310	1.252316	Protein modification process
1.30E-109	1636	1974	1.373018	Cellular macromolecule localization
1.37E-108	1624	1960	1.372683	Cellular protein localization
4.52E-99	2775	3660	1.256094	Cellular component biogenesis
2.22E-92	1853	2332	1.316398	Cellular response to stress
1.55E-91	1611	1989	1.341841	Establishment of protein localization
7.74E-88	2312	3018	1.269138	Establishment of localization in cell
8.04E-87	1515	1867	1.344338	Protein transport
2.20E-86	2287	2987	1.268444	Catabolic process
2.37E-86	2000	2566	1.29126	Cellular catabolic process
8.78E-85	1602	1997	1.328999	Cell cycle
3.45E-81	3390	4675	1.201319	Organic cyclic compound biosynthetic process
4.82E-81	3274	4498	1.205867	Heterocycle biosynthetic process

**Table 3:** The top 20 GO enrichment analysis of cellular component in castration-resistant prostate cancer. FDR: false discovery rate; GO: gene ontology

Enrichment FDR	N genes	Pathway genes	Fold enrichment	Pathway
4.25E-254	3948	4885	1.338914	Nuclear lumen
3.83E-241	3671	4519	1.345805	Nucleoplasm
1.01E-96	1498	1816	1.366584	Mitochondrion
4.12E-85	1278	1540	1.374834	Catalytic complex
8.17E-83	3008	4085	1.219905	Organelle membrane
2.19E-77	1156	1391	1.3768	Microtubule cytoskeleton
3.48E-64	1102	1352	1.350346	Organelle envelope
3.48E-64	1102	1352	1.350346	Envelope
1.06E-60	745	868	1.421925	Microtubule-organizing center
2.50E-59	888	1067	1.37876	Nucleolus
1.63E-55	1871	2504	1.237883	Cytoskeleton
1.63E-55	1102	1379	1.323907	Nuclear protein-containing complex
1.80E-54	713	839	1.407887	Transferase complex
1.23E-53	577	658	1.452747	Centrosome
8.85E-47	723	872	1.373606	Mitochondrial envelope
1.42E-43	452	512	1.462543	Mitochondrial matrix
9.46E-42	700	854	1.357939	Intracellular protein-containing complex
1.52E-41	655	792	1.370113	Ribonucleoprotein complex
2.82E-41	676	822	1.362433	Mitochondrial membrane
2.35E-38	748	932	1.329615	Nuclear body

**Table 4:** The top 20 GO enrichment analysis of molecular functions in castration-resistant prostate cancer. FDR: false discovery rate; GO: gene ontology

Enrichment FDR	N genes	Pathway genes	Fold enrichment	Pathway
2.65E-112	3287	4371	1.245831	Nucleic acid binding
3.29E-101	1778	2197	1.340732	Enzyme binding
1.44E-95	1537	1873	1.359491	RNA binding
1.77E-72	1839	2382	1.279028	Nucleoside phosphate binding
1.88E-72	1838	2381	1.27887	Nucleotide binding
3.17E-64	1977	2620	1.250102	Anion binding
1.14E-61	2049	2739	1.239339	Small-molecule binding
8.22E-60	1627	2122	1.27023	Purine nucleotide binding
2.34E-59	1925	2565	1.243322	Transferase activity
1.09E-58	1625	2124	1.267474	Ribonucleotide binding
1.42E-58	1613	2107	1.268265	Purine ribonucleotide binding
1.13E-57	1560	2033	1.27124	Purine ribonucleoside triphosphate binding
1.48E-57	3276	4636	1.170687	Metal ion binding
7.00E-57	3331	4726	1.167673	Cation binding
1.17E-48	1337	1743	1.270791	Adenyl nucleotide binding
1.45E-47	1325	1730	1.268849	Adenyl ribonucleotide binding
2.59E-46	1275	1662	1.270923	ATP binding
4.71E-42	658	794	1.372921	Kinase binding
1.07E-41	1817	2492	1.207945	Carbohydrate derivative binding
1.09E-40	1943	2689	1.197077	DNA binding

**Table 5:** The top 20 GO enrichment analysis of KEGG pathway in castration-resistant prostate cancer. FDR: false discovery rate; GO: gene ontology; KEGG: Kyoto Encyclopedia of Genes and Genomes

Enrichment FDR	N genes	Pathway genes	Fold enrichment	Pathway
4.62E-39	1163	1527	1.261772	Metabolic pathways
3.28E-19	302	363	1.37829	Amyotrophic lateral sclerosis
3.61E-18	314	383	1.358223	Alzheimer disease
4.34E-18	393	495	1.315308	Herpes simplex virus 1 infection
9.72E-18	257	306	1.3914	Huntington disease
1.85E-17	377	475	1.314886	Pathways of neurodegeneration
2.70E-17	214	249	1.423819	Salmonella infection
4.50E-17	131	141	1.539191	Ubiquitin-mediated proteolysis
1.41E-16	200	232	1.428178	Thermogenesis
1.53E-16	225	266	1.401332	Parkinson disease
3.51E-16	214	252	1.406868	Endocytosis
5.28E-15	150	169	1.470431	Protein processing in endoplasmic reticulum
1.79E-14	116	126	1.525203	Cell cycle
2.20E-14	406	530	1.269084	Pathways in cancer
5.98E-14	120	132	1.506078	Spliceosome
8.07E-14	205	246	1.380572	Shigellosis
1.45E-13	121	134	1.495963	Ribosome
3.74E-13	141	161	1.450887	MicroRNAs in cancer
1.07E-12	186	223	1.38181	Chemical carcinogenesis
1.29E-12	125	141	1.468693	Autophagy

**Table 6:** Key hub genes identified in the network analysis of castration-resistant prostate cancer (CRPC).

Gene name	Function	Network category
CD9	Cell adhesion and communication	Cellular communication
UBE2G2	Ubiquitination and protein degradation	Cellular communication
TRA2B	RNA splicing and regulation	Cellular communication
MT-CO1	Mitochondrial respiration	Metabolic reprogramming
ATP5-MC2	ATP synthesis	Metabolic reprogramming
CHD7	Chromatin remodeling	DNA repair and chromatin remodeling
CHD8	Epigenetic regulation	DNA repair and chromatin remodeling
CDC25B	Cell cycle regulation	DNA repair and chromatin remodeling
GAPDH	Glycolysis and energy metabolism	Stress response and protein homeostasis
HSP90AA1	Protein folding and stress response	Stress response and protein homeostasis

These findings provide a comprehensive molecular landscape of castration-resistant prostate cancer, revealing key pathways and gene networks that contribute to tumor progression, therapeutic resistance, and potential precision medicine strategies. The identified critical regulators play pivotal roles in various hallmarks of cancer, including sustained proliferative signaling, metabolic reprogramming, genomic instability, and evasion of cell death. These insights offer valuable opportunities for the development of targeted therapeutic approaches and precision medicine strategies for the management of castration-resistant prostate cancer.

## Discussion

This study provides a comprehensive molecular characterization of castration-resistant prostate cancer progression by examining DEGs between normal prostate epithelial cells and CRPC cells. The findings highlight significant molecular alterations associated with CRPC, particularly in metabolic reprogramming and cell cycle regulation. These results offer valuable insights into potential therapeutic targets and biomarkers that could aid in early detection, prognosis, and treatment of CRPC. To further investigate these molecular changes, differential expression analysis and gene network analysis were performed. Figure 2 presents a heatmap of the 10 most significantly differentially expressed genes, highlighting genes with the highest magnitude of transcriptional change between PNT2 and PC3M cells based on statistical significance. However, while differential gene expression analysis identifies genes with substantial expression differences, it does not necessarily highlight genes that are functionally central in cancer progression. Therefore, a gene network analysis was conducted to identify key regulatory genes based on their connectivity within biological pathways. The hub gene analysis revealed critical regulators involved in cellular communication, metabolic reprogramming, chromatin remodeling, and stress response mechanisms. These key molecular pathways and signaling networks play pivotal roles in driving the progression and therapeutic resistance of CRPC, making them promising targets for the development of novel treatment strategies. Although the current study utilizes immortalized cell lines, which may not fully capture the multifaceted nature of patient-derived tumors, future investigations will aim to validate the top candidate genes using CRISPR-Cas9 knockout techniques. This will be followed by transcriptomic analyses, in vivo experimentation, and clinical correlation studies to evaluate the relevance of these genes as potential therapeutic targets and biomarkers.

Dysregulation of key molecular pathways in CRPC

Functional enrichment analysis revealed that biological processes such as cell cycle regulation, metabolic adaptation, intracellular signaling, and macromolecule biosynthesis were significantly altered in CRPC cells. The GO enrichment analysis identified pathways related to cell cycle progression, intracellular transport, and protein localization, which are critical for maintaining the proliferative capacity of CRPC cells (Figure 3). The observed upregulation of cell cycle-related genes such as CDC25B and GTSE1 (Figure 7d) suggests that cell cycle dysregulation plays a key role in castration resistance, consistent with previous studies on CRPC progression [24-27].

Moreover, KEGG pathway analysis demonstrated significant enrichment in ubiquitin-mediated proteolysis, autophagy, and protein processing pathways, indicating that CRPC cells employ alternative mechanisms for protein degradation and cellular homeostasis in response to metabolic and therapeutic stress (Figure 6). These findings align with emerging evidence suggesting that alterations in protein degradation pathways contribute to therapy resistance in prostate cancer [28].

Cellular communication and signal transduction mechanisms in CRPC

Analysis of the gene interaction network depicted in Figure 7b revealed key genes with pivotal roles in cellular communication and signal transduction pathways, including CD9, UBE2G2, and TRA2B. These genes modulate critical cellular processes such as cell-cell adhesion, cell migration, and the ubiquitin-proteasome system, thereby enhancing the survival, proliferation, and metastatic potential of castration-resistant prostate cancer cells [29]. The protein CD9 is involved in controlling exosome communication and the epithelial-to-mesenchymal transition (EMT), processes that contribute to cancer progression. Similarly, the enzyme UBE2G2 is associated with protein degradation pathways, affecting tumor growth and therapy resistance in androgen-independent prostate cancer [30]. Additionally, the splicing regulator TRA2B has been linked to alternative splicing that drives cancer pathways and metastasis in various cancer types, including prostate cancer. Disruptions or dysregulations in these signaling pathways can have a significant influence on the progression, invasiveness, and aggressiveness of CRPC, making them potential therapeutic targets for intervention and management of this advanced prostate cancer subtype [31].

Metabolic reprogramming and mitochondrial involvement in CRPC

One of the most striking findings was the differential expression of mitochondrial genes, including MT-CO1, MT-CYB, MT-ND4, and MT-ATP6, which were significantly upregulated in PC3M cells. Mitochondrial dysfunction has been increasingly recognized as a hallmark of cancer, particularly in CRPC, where metabolic reprogramming enables tumor cells to thrive under nutrient-deprived and hypoxic conditions [32,33]. The upregulation of these mitochondrial genes suggests that CRPC cells rely more heavily on oxidative phosphorylation (OXPHOS), which has been proposed as a metabolic adaptation in androgen-independent prostate cancer [34,35]. Recent studies have demonstrated that detrimental alterations in mitochondrial complex I and IV genes, including MT-ND4 and MT-CO1, are linked to enhanced oxidative phosphorylation activity and increased aggressiveness in high-grade and treatment-resistant prostate cancers. Additionally, such dysregulation appears to promote elevated ATP production and reactive oxygen species generation, both of which contribute to resistance against apoptosis and hormonal therapy [36]. These findings align with the emerging concept that mitochondrial remodelling is central to the progression of castration-resistant prostate cancer, suggesting that targeting mitochondrial metabolism either through OXPHOS inhibitors or modulation of mitochondrial gene expression may represent a promising therapeutic approach for suppressing tumor growth and overcoming drug resistance [37,38].

Chromatin remodeling and DNA repair pathways in CRPC

Dysregulation of chromatin remodelling and DNA repair genes, including CHD7, CHD8, and CDC25B, significantly contributes to the progression of castration-resistant prostate cancer. Alterations in the expression or function of these genes can lead to aberrant gene expression patterns and compromised genomic integrity, ultimately driving the advancement of CRPC (Figure 7d) [39]. Recent studies have highlighted the involvement of CHD7 in the transcriptional regulation of cancer-associated genes, with evidence suggesting that its dysregulation may contribute to altered chromatin architecture and oncogenic signaling in advanced prostate malignancies [40]. CHD8 has also been implicated in CRPC, where its increased nuclear expression correlates with poor prognosis and enhanced metastatic potential, supporting its role in disease progression [41]. Furthermore, CDC25B, a cell cycle phosphatase frequently overexpressed in high-grade prostate tumors, has been shown to act as a co-activator of the androgen receptor (AR), thereby promoting AR-mediated transcription even under androgen-deprived conditions [42]. Targeting these critical chromatin remodelling and DNA repair pathways, particularly in tumors with impaired DNA repair mechanisms, aligns with the success and potential of therapies such as PARP inhibitors in the management of prostate cancer [43,44]. Exploiting the vulnerabilities of CRPC cells resulting from dysfunctional DNA repair could provide novel therapeutic avenues to improve patient outcomes.

Stress response and protein homeostasis in CRPC

Genes central to stress responses and protein homeostasis, such as GAPDH and HSP90AA1, were identified as key players (Figure 7e). These genes play a crucial role in protecting CRPC cells from stress-induced damage and apoptosis, thereby contributing to therapy resistance [45,46]. Recent research has shown that increased expression of GAPDH in cancer cells improves their survival under low oxygen conditions and contributes to drug resistance. This is achieved through GAPDH's interaction with the AKT signalling pathway, which promotes cell proliferation and tumor growth [47]. Furthermore, HSP90AA1, a molecular chaperone, is vital for preserving protein balance in CRPC cells. Its heightened expression is linked to unfavourable prognosis and has been implicated in stabilising client proteins that are crucial for cancer cell survival under stressful conditions [48]. Modulating these stress response and protein homeostasis mechanisms could potentially enhance the effectiveness of conventional therapies by disrupting the critical survival pathways in castration-resistant prostate cancer cells [49].

## Conclusions

This study provides a comprehensive molecular analysis of castration-resistant prostate cancer (CRPC) by comparing gene expression profiles between normal prostate epithelial cells (PNT2) and CRPC cells (PC3M), revealing significant alterations in cell cycle regulation, metabolic pathways, DNA repair mechanisms, and stress response networks. These changes underscore the complexity of CRPC biology and contribute to the progression and therapeutic resistance of the disease. The identified molecular alterations offer insights into potential therapeutic targets and biomarkers for early detection, prognosis, and treatment strategies. Highlighting the need for targeted therapeutic approaches based on detailed molecular profiles, this research emphasizes that further investigation is crucial to translate these findings into clinical practice, aiming to improve outcomes for patients with CRPC.
